# Nitrogen-Doped Porous Carbon Materials Derived from Graphene Oxide/Melamine Resin Composites for CO_2_ Adsorption

**DOI:** 10.3390/molecules26175293

**Published:** 2021-08-31

**Authors:** Like Ouyang, Jianfei Xiao, Housheng Jiang, Shaojun Yuan

**Affiliations:** Low-Carbon Technology & Chemical Reaction Engineering Lab, College of Chemical Engineering, Sichuan University, Chengdu 610065, China; Like.ouyang@scu.edu.cn (L.O.); JianfXiao@163.com (J.X.); jianghousheng819@163.com (H.J.)

**Keywords:** nitrogen-doped, porous carbon, melamine resin, graphene oxide, CO_2_ adsorption

## Abstract

CO_2_ adsorption in porous carbon materials has attracted great interests for alleviating emission of post-combustion CO_2_. In this work, a novel nitrogen-doped porous carbon material was fabricated by carbonizing the precursor of melamine-resorcinol-formaldehyde resin/graphene oxide (MR/GO) composites with KOH as the activation agent. Detailed characterization results revealed that the fabricated MR(0.25)/GO-500 porous carbon (0.25 represented the amount of GO added in wt.% and 500 denoted activation temperature in °C) had well-defined pore size distribution, high specific surface area (1264 m^2^·g^−1^) and high nitrogen content (6.92 wt.%), which was mainly composed of the pyridinic-N and pyrrolic-N species. Batch adsorption experiments demonstrated that the fabricated MR(0.25)/GO-500 porous carbon delivered excellent CO_2_ adsorption ability of 5.21 mmol·g^−1^ at 298.15 K and 500 kPa, and such porous carbon also exhibited fast adsorption kinetics, high selectivity of CO_2_/N_2_ and good recyclability. With the inherent microstructure features of high surface area and abundant N adsorption sites species, the MR/GO-derived porous carbon materials offer a potentially promising adsorbent for practical CO_2_ capture.

## 1. Introduction

Carbon dioxide (CO_2_) emissions are generally recognized as the leading cause of global warming. Recently, the United Nations Environment Program’s (i.e., UNEP) annual Emissions Gap Report 2020 reported that global greenhouse gas (GHS) emissions continued to increase and reached a record high of 52.4 ± 5.2 Gt CO_2_e without land-use change (LUC) emissions and 59.1 ± 5.9 Gt CO_2_e with LUC in 2019. Fossil CO_2_ emissions dominate total GHS emissions with 65% and reached a record 38.0 ± 1.9 GtCO_2_ on the basis of preliminary data [1]. Carbon capture and storage (CCS) is broadly regarded as one of the most effective techniques to reduce CO_2_ emission [2,3,4]. Among the various technologies developed, adsorption including pressure swing adsorption (PSA) and temperature swing adsorption (TSA) by using porous solid materials have attracted tremendous attentions due to their high capture efficiency, low capital input and operation cost, low energy consumption and high stability [5].

Over the past decades, different porous solid materials such as carbon materials, zeolite, metal-organic frameworks, porous polymers and their composite materials have been investigated as CO_2_ adsorbents [6,7,8,9]. Particularly, porous carbon materials (PCM) show great application potential in CO_2_ capture because of their low cost, abundant source, excellent CO_2_ adsorption performance and physicochemical stability [10,11,12]. It has been generally recognized that PCM with large specific surface area and adequate pore structure has high adsorption capacity for CO_2_. The introduction of nitrogen atoms into the framework of PCM will further enhance CO_2_ capture and selectivity by the interaction between basic nitrogen sites and acidic CO_2_ molecules [13,14,15]. Moreover, compared with nonpolar N_2_ molecules, the polar CO_2_ molecules were preferred to be adsorbed by electronegative N species, thus enhancing the selectivity for CO_2_ capture and separation of CO_2_ from N_2_/CO_2_ gas mixture. Therefore, different methods have been developed to prepare the nitrogen-doped porous carbon material (NPCM). 

Up to now, two commonly used ways have been reported to synthesize the NPCM, one is direct pyrolysis and activation of nitrogenous organic precursors, the other is to introduce nitrogen element by using nitrogenous activating agents during the activation at elevated temperatures [16,17,18,19]. Because the nitrogen is derived from the precursor itself, the former method is more conducive to the formation of abundant nitrogen species in the framework of the NPCM. By well designing of precursor materials, precisely controlling of pyrolysis and activation temperature, carefully selecting of activating agents and reaction atmosphere. Thus, the NPCM with high specific surface area, well-defined microstructure and active surface nitrogen species (e.g., pyridinic, pyrrolic, quaternary and oxidized nitrogen) can be achieved for enhanced CO_2_ capture performance. Both inorganic such as ammonium [20] and sodium amide [21] and organic nitrogen-containing materials such as urea [22], dopamine [23], pyrrole [15], melamine [24], ethylenediamine [25], benzenediamine [26], dicyandiamide [27], hexamethylenetetramine [28], benzimidazole [29], carbazole [30], cyanuric chloride [31], polyacrylonitrile [32], polyimide [33], polyindole [34] and chitosan [35] have been used as nitrogen source of NPCM with a content range of 0.23–31.70 wt.%. Generally, pyrolysis and activation of nitrogenous precursors at low temperature are favorable for nitrogen retention. Yue et al. used D-glucose as carbon source and urea as nitrogen source to synthesize NPCMs, and found that the nitrogen content decreased from 11.81 to 4.59 wt.% upon increasing temperature from 600 to 700 °C [36]. Tseng et al. reported the similar findings, that the nitrogen content decreased from 3.0 to 1.0 wt.% at an elevated temperature on the melamine-modified phenol-formaldehyde resin-based NPCM [37]. In our previous study, the decrease in nitrogen content from 10.21 to 7.07 wt.% with the increase in temperature from 400 to 700 °C was also observed on the dicyandiamide-based NPCM [38]. However, upon being activated at a low temperature, it is difficult for the NPCM to form a well-developed pore structure, high specific surface area and pore volume, which is also a critical factor for the CO_2_ adsorption. Generally, the micropores enhance the interaction between CO_2_ molecules and adsorbent, whilst the mesopores can improve the utilization efficiency of the nitrogenous adsorption sites and surface area and accelerate the diffusion of CO_2_ in the pore channels. Therefore, it is of great importance to optimize pyrolysis and activation temperature for balancing the surface area and nitrogen content.

The selection of activating agents such as KOH, Na_2_CO_3_ and ZnCl_2_ and its adding ratio to carbon precursor also have significant effect on the pore structure, surface area and nitrogen content of the NPCM, and then affect the CO_2_ capture performance [39,40]. For instance, Shao et al. found that the NPCM prepared at 800 °C using KOH as activating agent had the highest surface area of 3542 m^2^/g with nitrogen content of 0.75 wt.%, the NPCM prepared at 700 °C with ZnCl_2_ had the highest nitrogen content of 7.27 wt.% and highest CO_2_/N_2_ adsorption selectivity of 42.6, whilst the NPCM prepared at 700 °C with KOH had the nitrogen content of the highest CO_2_ uptake of 5.45 mmol·g^−1^ [31]. By using phenolic resin (PR) as carbon precursor and NaNH_2_ as both activation agent and nitridation reagent, Wang et al. found that the nitrogen content for the NPCM increased from 1.56 to 5.94 wt.% with the increase in the NaNH_2_/PR ratio from 1 to 4 and the NPCM with the nitrogen content of 4.25 wt.% has the highest surface area of 1432 m^2^/g and CO_2_ uptake of 4.64 mmol·g^−1^ at 25 °C at 1 atm [17].

Melamine, as a nitrogen-rich organic molecule and low-cost raw material in the chemical industry, has been extensively employed in the preparation of NPCM for the CO_2_ capture [41]. Lee et al. prepared melamine-based NPCM with CO_2_ capture capacity of 1.77 mmol·g^−1^ and very high CO_2_/N_2_ adsorption selectivity of 100 at 25 °C at 1 atm [42]. Wu et al. reported a CO_2_ adsorption capacity of 3.64 mmol·g^−1^ at 25 °C and 100 kPa using the activated dopamine-melamine copolymer [24]. Rehman and Park fabricated glucose-melamine-based activated carbon with a high CO_2_ adsorption capacity of 4.95 mmol·g^−1^ at 25 °C and 100 kPa [43]. These studies gave very detailed results for the CO_2_ adsorption behavior on melamine-based NPCM at low temperature (0–25 °C) or low pressure (0–100 kPa). However, few studies have been documented to study the CO_2_ adsorption on melamine-based NPCM at a relatively high temperature and high pressure, which is closer to the industrial conditions applied in CO_2_ capture.

Herein, the aim of the study is to prepare a novel NPCM derived from the composite material composed of melamine-resorcinol-formaldehyde resin and graphene oxide(MR/GO) for CO_2_ adsorption in a temperature range of 25–50 °C and pressure range of 0–500 kPa. The effects of pyrolysis temperature (400–700 °C) and the amount of GO added on the pore structure and surface features of NPCM were systematically investigated. The CO_2_ adsorption behaviors including adsorption isotherms, thermodynamics, kinetics and cyclic adsorption property were also investigated. The unique pore structure and doped nitrogen species for MR/GO-based NPCM significantly facilitated CO_2_ adsorption and selectivity, exhibited highly potential values in CO_2_ capture.

## 2. Materials and Methods

### 2.1. Materials

Melamine (AR), resorcinol (AR) and graphene oxide (GO, >99%) were purchased from Aladdin^®^ Co. (Shanghai, China). Formaldehyde (37% aqueous solution), ethanol (AR), and potassium hydroxide (KOH, 37%) were obtained from Chengdu Kelong Reagent Co. (Chengdu, China). Deionized water (18 MΩ·cm) was produced by an ultrapure water system from Ultrapure Technol. Co. (Chengdu, China).

### 2.2. Synthesis and Activation of GO/MR Composite

**Figure 1:**
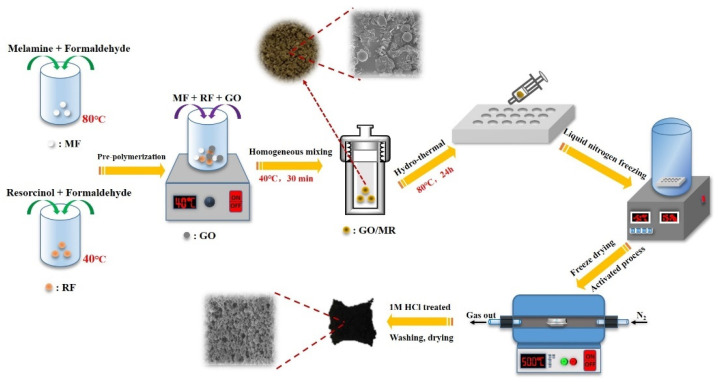
Schematic illustration of the synthesis process of nitrogen-rich porous carbon materials derived from GO/MR composite.

The synthesis process of GO/MR composite and derived NPCM is illustrated in Figure 1. Firstly, an aliquot 1.94 g of resorcinol and 2.85 g of formaldehyde aqueous solution were introduced into a 100 mL beaker A containing 15 mL ultra-pure water. The mixed solution was magnetically stirred until all the resorcinol was dissolved, then heated to 40 °C in a water bath and kept for 30 min. The mixture was a prepolymer of resorcinol and formaldehyde and named as the precursor RF solution. Meanwhile, an aliquot 2.22 g of melamine powder and 4.28 g of formaldehyde aqueous solution were poured into another beaker B (100 mL) containing 15 mL ultra-pure water. The mixed solution was magnetically stirred at 80 °C in a water bath until it changed from white liquid mixture to a transparent liquid, followed by cooling the resultant solution to 40 °C. This prepolymer of melamine and formaldehyde was named as the precursor MF solution. Finally, RF, MF and GO were mixed with magnetic stirring at 40 °C for 30 min until the solution became even, and then transferred to a hydrothermal synthesis reactor. The mass ratios of GO addition amount were 0, 0.250 and 0.375 wt.% of total mass (MF + RF + GO), respectively. The hydrothermal reaction was proceeded at 80 °C for 24 h to obtain the GO/melamine resin composites. After the reaction, the solid composite was filtrated out, washed with copious amounts of ultra-pure water and ethanol and frozen in liquid nitrogen prior to being freeze-dried for 24 h. The resulting dried composite was denoted as GO/MR composite.

To obtain GO/MR-derived NPCM with a high surface area and abundant pores, the activation was carried out under a nitrogen stream with KOH as activator. Briefly, 1 g of GO/MR was mixed with 2 g of KOH in ultra-pure water and then freeze-dried. Subsequently, the obtained solid was activated at 400–700 °C for 2 h with a ramp rate of 5 °C·min^−1^ and a N_2_ flow rate of 60 mL·min^−1^. After pyrolysis treatment, the carbon materials were washed with ethanol and ultra-pure water alternately until the pH of filtrate reached around 7, then dried in vacuum under 80 °C. The finally obtained carbon materials with activation temperature of 500 °C and 0.250 wt.% GO added was named GO(0.25)/MR-500.

### 2.3. Characterization

The morphology of all samples was analyzed by scanning electron microscope (SEM, JSM-7610F with an accelerating voltage of 15 kV) and transmission electron microscopy (TEM, Tecnai G2 F20 S-TWIN field emission TEM with operating acceleration voltage of 200 kV), respectively. The crystalline structure of the samples was measured by using X-ray diffractometer (XRD, DX2700, Fangyuan Instruments Co., Wenzhou, China) with Cu Ka radiation (k = 1.5418 Å). The Fourier transform infrared spectroscopy (FTIR) was collected in a range of 400–4000 cm^−1^ on a Perkin-Elmer FTIR spectrometer. Raman spectra were collected using a DXR Raman Microscope (Thermo Fisher Scientific Inc., Waltham, MA, USA) with excitation wavelength of 455 nm. The X-ray photoelectron spectroscopy (XPS, a Kratos Axis Ultra spectrometer) with a monochromatic Al Ka X-ray source (1486.6 eV photons, pass energy 20.0 eV) was used to analyze the surface structure of all samples. The specific surface area was calculated by using the Brunauer–Emmett–Teller (BET) method on a Micromeritics analyzer ASAP 2460.

### 2.4. Adsorption Experiment

The CO_2_ adsorption isotherms of all samples were measured at 25–50 °C and 0–500 kPa using a self-made static adsorption apparatus, as shown in Supplementary Materials, Figure S1. The detailed procedures are described in ESI Section S1.1. Nitrogen adsorption isotherms at 25 °C and 0–500 kPa were also measured to evaluate the CO_2_/N_2_ selectivity. For each measurement, the sample was degassed at 100 °C under a high vacuum for more than 12 h.

## 3. Results and Discussion

**Figure 2:**
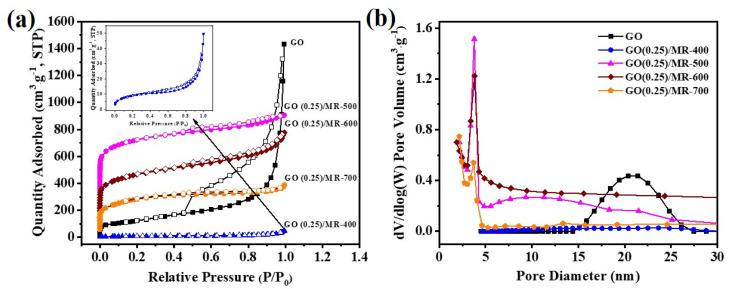
(**a**) N_2_ adsorption-desorption isotherms and (**b**) pore size distributions of GO and GO(0.25)/MR-derived nitrogen-rich porous carbon materials obtained at different activation temperature.

Figure 2a shows N_2_ adsorption-desorption isotherms and pore size distributions of GO and GO(0.25)/MR-derived porous carbon materials at different activation temperatures. The isotherms of GO and GO(0.25)/MR-derived carbon materials display type IV isotherms according to IUPAC classification, which indicates the existence of micropores and mesopores. A slow climb for N_2_ adsorption at low relative pressure (P/P_0_ < 0.01) and a steep rise in N_2_ adsorption at high relative pressure (P/P_0_ > 0.4) are observed. In addition, there is a large hysteresis loop between 0.4 and 0.9 of the relative pressure P/P_0_, suggesting the abundant mesopores for GO. When the activation temperature is 500–700 °C, all the isotherms of carbon materials show mix of type I and type VI isotherms, which indicates that these carbon materials process abundant micropores and mesopores. As shown in Table 1, GO has a BET surface area of 450 m^2^·g^−1^, pore volume of 2.20 cm^3^·g^−1^ and mean pore size of 20.8 nm. After in situ polymerization of melamine-resorcinol resin (MR) on GO, the BET surface area, pore volume and mean pore size of GO(0.25)/MR decreased to 6.7 m^2^·g^−1^, 0.003 cm^3^·g^−1^ and 8.5 nm, respectively. The results indicated the pore filling and surface coverage of MR on the GO. With the activation temperature at 400 °C and 500 °C, the BET surface area increased to 32 m^2^·g^−1^ for GO(0.25)/MR-400 and the highest BET surface area of 1264 m^2^·g^−1^ was obtained for GO(0.25)/MR-500, respectively. As shown in Figure 2b, it is interesting to note that when the activation temperature is up to 500 °C, a large number of micropores with a narrow distribution are formed and partial mesopores still exist, leading to a mean pore size of 3.8 nm. However, with further increase in activation temperature to 600 and 700 °C, the BET surface area decreases to 972 and 790 m^2^·g^−1^, respectively. Meanwhile, the number of micropores also decreases. Furthermore, the effect of adding amount for GO on the pore structure was also studied and all the samples also display type IV isotherms (Figure S2). As shown in Table 1, the MR-500 without GO has a BET surface area of 204 m^2^·g^−1^, pore volume of 0.10 cm^3^·g^−1^ and mean pore size of 3.55 nm. With the increase in GO from 0.25 wt.% to 0.375 wt.%, the BET surface area and the pore volume decreases from 1264 to 784 m^2^·g^−1^ and from 0.47 to 0.39 cm^3^·g^−1^. These results suggest that the appropriate addition of GO is beneficial to the formation of pores and increases the BET surface area.

**Figure 3:**
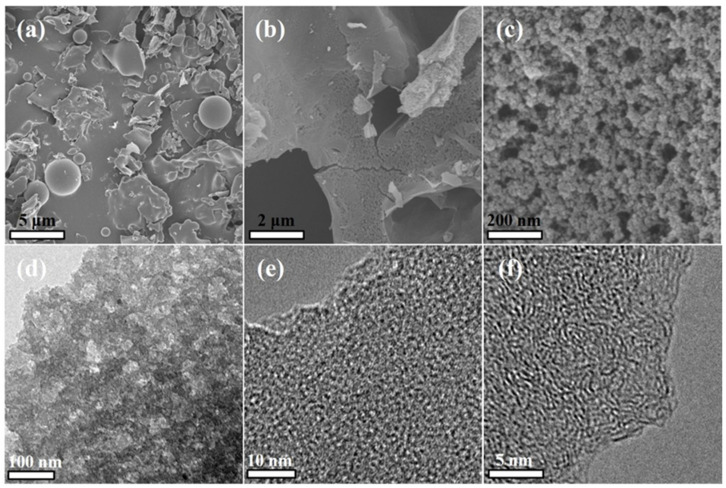
SEM images of (**a**) GO(0.25)/MR and (**b**,**c**) GO(0.25)/MR-500, (**d**) TEM image and (**e**,**f**) high-resolution TEM image of GO(0.25)/MR-500.

The morphology of GO(0.25)/MR and GO(0.25)/MR-500 was characterized by using SEM and TEM. As shown in Figure 3a, the GO(0.25)/MR without activation exhibits a heterogeneous surface morphology with a compact stacked structure, which indicates the successful polymerization of MR on GO. Moreover, a small amount of spherical MR particles with different sizes is supported on the surface of GO(0.25)/MR composite. From the SEM images of GO(0.25)/MR-500 (Figure 3b,c), it is clearly shown that a few ant nest-like nanoscale pores with uniform distribution have been generated. This result is because KOH uniformly adheres to the surface of the composite, which is then melted, carburized and vaporized in the process of high-temperature carbonization, resulting in the uniform pores. The porous structure is also revealed by the TEM images (Figure 3d). It is very interesting that wormlike morphology with the formation of rich micropores can be observed from the high-resolution TEM (Figure 3e,f) and this is originated from the random assembling of graphene layers.

**Figure 4:**
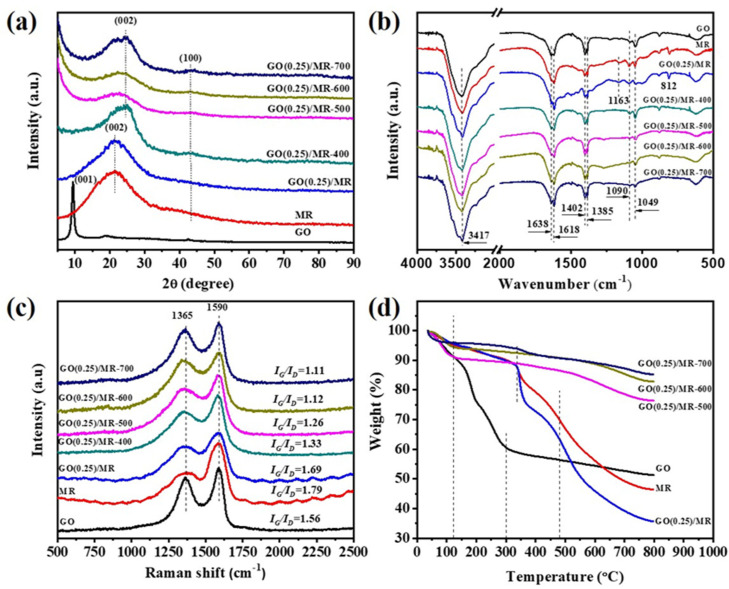
(**a**) XRD, (**b**) FTIR, (**c**) Raman patterns and (**d**) TGA curves of GO, MR, GO(0.25)/MR and GO(0.25)/MR-derived nitrogen-rich porous carbon materials obtained at different activation temperature.

Figure 4a shows the XRD patterns of precursors, GO(0.25)/MR and its derived carbon materials. For GO(0.25)/MR, the broad peaks at 2θ = 22.4° and 43.9° belong to the (002) and (100) diffraction patterns of amorphous graphitic carbon. After activation, the peak assigned to (002) diffraction pattern shifts to 24.2° and peak broadening occurs. This is probably due to the destruction of graphitized structure by KOH in the process of high temperature activation and confirms the amorphous nature of GO(0.25)/MR-derived carbon materials, which is consistent with the results reported in the literature [44]. Figure 4b shows the FTIR spectra of all samples. The peaks at around 3417 cm^−1^ and 1618/1638 cm^−1^ can be assigned to the stretching vibration of –OH of GO and adsorbed H_2_O, respectively [45,46]. The absorption at 1402, 1385 and 1049/1090 cm^−1^ can be attributed to C=C stretching vibration, tertiary alcoholic C–OH bending and C–O stretching vibration, respectively [47,48,49]. Compared with GO, new peaks at 1557, 1490, 1163 and 812 cm^−1^ appear on GO(0.25)/MR composite, which are associated to N–H in-plane deformation vibration, aromatic C–C stretching vibration, C–N stretching vibration and s-triazine rings in the carbon matrix [50,51]. However, these peaks disappear after activation of GO(0.25)/MR at high temperature and all the GO(0.25)/MR-derived carbon materials show similar FTIR spectra with GO. Raman spectra (Figure 4c) show two clear peaks at 1365 cm^−1^ (D band) and 1590 cm^−1^ (G band) for all samples. The D band and G band are related to the defects or disorder in carbon and the vibration of sp^2^ hybridized carbon atoms in graphitic layer. Generally, the I_D_/I_G_ value increases with the increase in activation temperature, indicating that more defects were generated. Generally, the adsorption capacity is correlated with I_D_/I_G_ ratio and increases with increasing I_D_/I_G_ ratio. The thermal stability of GO(0.25)/MR-derived carbon materials was also studied by using TGA and shown in Figure 4d. Compared with GO(0.25)/MR, the GO(0.25)/MR-derived carbon materials have good thermal stability. At the temperature below 120 °C, only a small weight loss ascribed to the water adsorbed on surface is observed. Even when the temperature is up to 500 °C, the weight loss of GO(0.25)/MR-derived carbon material (i.e., GO(0.25)/MR-500) is smaller than 15%, while the weight loss of GO(0.25)/MR is more than 40%.

**Figure 5:**
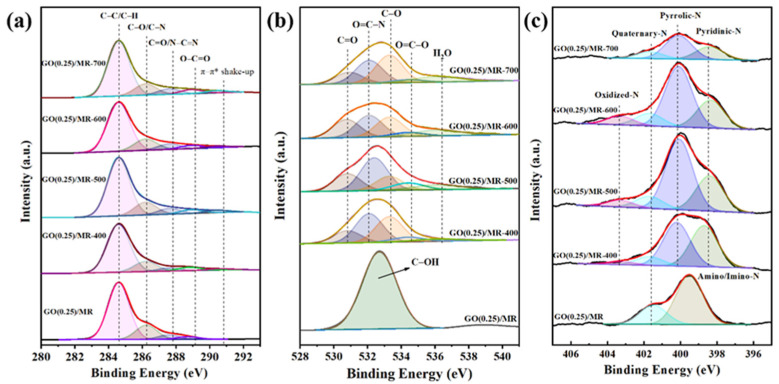
High resolution (**a**) C 1s, (**b**) O 1s and (**c**) N 1s XPS spectra of GO(0.25)/MR and GO(0.25)/MR-derived nitrogen-rich porous carbon materials obtained at different activation temperature.

XPS was used to characterize the chemical information of GO(0.25)/MR composite and its derived carbon material. The wide XPS spectra (Figure S3) of all samples exhibited three peaks at 283.8, 400.2 and 534.4 eV, corresponding to C1s, N1s and O1s, respectively. Table 1 summarizes the quantitative data of surface element composition. The nitrogen content of GO(0.25)/MR composite was 7.30 wt.%, which proved that the nitrogen of melamine has been successfully doped into the composite. After activation in the temperature range at 400–600 °C, the nitrogen contents only showed a slight decrease and stayed above 6.5 wt.%. However, with the further increase in temperature to 700 °C, the nitrogen content decreased to 3.42 wt.%. Compared with the effect of activation temperature, the added amount of GO has little effect on the nitrogen content. The oxygen content for all activated samples at high temperature showed a significant decrease. The content loss of nitrogen and oxygen could be attributed to the relatively higher activity of nitrogen and oxygen compared to carbon under the activation in N_2_ atmosphere at high temperature. Figure 5a shows the high-resolution C 1s spectra for all samples and peaks centered at 284.6, 286.3, 287.6 and 288.7 eV belong to the C−C/C−H, C−O/C−N, C=O/N−C=N and O=C–O bonds [52,53,54]. The peak at 290.4 eV appearing in the C1s spectrum of activated samples corresponded to the π-π* shake-up peak due to the π–π interactions of conjugated aromatic structure [55]. Figure 5b shows the deconvolution of O 1s spectra and only one symmetric peak at 532.7 eV could be obtained for unactivated GO(0.25)/MR. This peak should be attributed to the oxygen species of C−OH. The deconvolution of O 1s spectra for activated samples at 400–700 °C results five different peaks with BEs at 530.8, 532.1, 533.3, 534.3 and 536.1 eV, corresponding to C=O, O=C−N, C−O, O=C–O and adsorbed water, respectively [56]. As shown in Figure 5c, the deconvoluted N 1s spectrum for unactivated GO(0.25)/MR results in two peaks at 399.5 and 401.5 eV, which can be attributed to amino/imino nitrogen and quaternary nitrogen, respectively [57,58]. After activation at 400−600 °C, the peak of amino/imino-N disappeared and three new peaks at 398.7, 400.1 and 403.2 eV appeared, attributable to pyridinic-N, pyrrolic-N and oxidized-N, respectively [59,60]. When further raising the activation temperature to 700 °C, the peak of oxidized-N disappeared. Table S1 gives the relative content of each kind of nitrogen and high relative content of pyrrolic-N and pyridinic-N were obtained for all activated samples. Because the pyrrolic-N donates two electrons to the p-system and pyridinic-N donates one electron to aromatic p-system, both of them exhibit Lewis basic character and have strong attraction for CO_2_ (a Lewis acid) capture [35]. Moreover, it was found that the GO(0.25)/MR-500 shows highest relative content of pyrrolic-N with 56.6%, which would further promote the capture of CO_2_ due to the stronger interactions that occur between pyrrolic-N and CO_2_ molecules [61].

**Figure 6:**
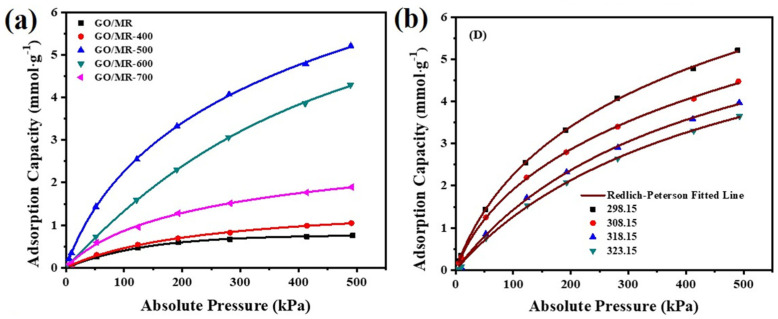
(**a**) CO_2_ adsorption isotherms at 298.15 K for GO(0.25)/MR and GO(0.25)/MR-derived, nitrogen-rich porous carbon materials obtained at different activation temperature. (**b**) Redlich–Peterson isotherm models on experimental CO_2_ adsorption at different temperature for GO(0.25)/MR-500.

Figure 6a shows the CO_2_ adsorption isotherms of GO(0.25)/MR composite and its derived carbon materials with different activation temperatures. Table 1 lists the static equilibrium adsorption capacity of CO_2_ at 298.15 K and 100/500 kPa. The adsorption capacity of all the samples increases with the increasing of pressure. Among them, GO(0.25)/MR-500 exhibits the best performance for CO_2_ adsorption with a capacity of more than 2.27/5.21 mmol·g^−1^ at 100/500 kPa. However, the GO(0.25)/MR without activation only has CO_2_ adsorption capacity of 0.40/0.76 mmol·g^−1^ at 100/500 kPa. With the increase in the activation temperature from 500 °C to 600 °C and 700 °C, the CO_2_ adsorption capacity of GO(0.25)/MR-600 and GO(0.25)/MR-700 decrease to 1.33/4.29 mmol·g^−1^ and 0.87/1.89 mmol·g^−1^ at 100/500 kPa, respectively. The MR-500 without GO and GO(0.375)/MR-500 has CO_2_ adsorption capacity of 0.19/0.49 mmol·g^−1^ and 0.74/2.81 mmol·g^−1^ at 100/500 kPa, respectively. This trend agrees well with the surface area and micropore volume of samples, which indicates that the CO_2_ adsorption capacity would increase with increasing surface area and micropore volume.

To understanding the CO_2_ adsorption mechanism of GO(0.25)/MR-500, the Langmuir, Freundlich, and Redlich–Peterson models were applied to simulate the CO_2_ adsorption at temperatures of 298.15–323.15 K (Figure 6b and  Figure S3a,b) and the fitting parameters are summarized in Table 2. Clearly, the Redlich––Peterson model provides the best fitting for experimental data at 298.15–313.15 K, indicating that the adsorption of CO_2_ can be explained by the micropore filling mechanism by multi-layer adsorption with both the physical and chemical adsorption. However, the best model tends toward Langmuir rather than Freundlich at elevated temperatures, revealing the mono-layer adsorption of CO_2_ on GO(0.25)/MR-500 at 323.15 K. This is also the main reason why the adsorption decreases with the increase in temperature. Hence, both the micropore volume and properties of the surface have strong effects on the CO_2_ adsorption capacity at low temperatures, while the role of the micropore structure gradually weakened and the properties of surface play dominant role influencing the CO_2_ adsorption capacity at high temperature. This is closely related to the chemical state and exposed content of N-doping sites at the surface.

**Figure 7:**
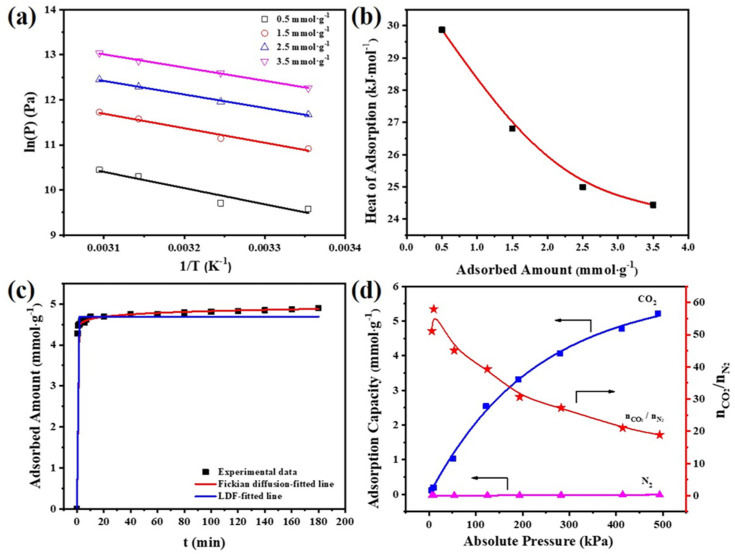
(**a**) Curves of ln(P) versus (1/T), (**b**) isosteric heat of adsorption at different CO_2_ adsorption capacities, (**c**) CO_2_ adsorption kinetics and (**d**) adsorption isotherms of N_2_ and CO_2_ at 298.15 K for GO(0.25)/MR-500.

The isosteric heat of adsorption (Qst) was calculated based on the isotherms at 298.15–323.15 K by employing the Clausis–Claperyon equation, and the plots of ln(P) as a function of 1/T are shown in Figure 7a. The Qst is obtained by linear fitting of the data, and its plot as a function of CO_2_ uptake for GO(0.25)/MR-500 is shown in Figure 7b. All the values of Qst are lower than 30 kJ·mol^−1^ (Table S2), indicating a physical adsorption process for GO(0.25)/MR-500 (Qst > 30 kJ·mol^−1^ for chemical adsorption). The results are similar with adsorption of CO_2_ on other carbon adsorbents. Moreover, the value of Qst decreases from 29.88 to 24.43 kJ·mol^−1^ with the increase in CO_2_ uptake from 0.5 to 3.5 mmol·g^−1^, revealing the gradual decrease in the interaction between CO_2_ and GO(0.25)/MR-500 and the surface heterogeneity of GO(0.25)/MR-500.

As shown in Figure 7c, very fast kinetics of CO_2_ adsorption were observed on GO(0.25)/MR-500, which reached 90 within 2 min and 97% within 10 min, respectively. In addition, the Fickian diffusion model was found more accurate to describe the kinetics of CO_2_ adsorption on GO(0.25)/MR-500 than the LDF model in Supplementary Figure S5, and the results are listed in Table S3. The diffusion time constant and equilibrium absorption capacity for CO_2_ is calculated to be 2.11 min^−n^ and 4.87 mmol∙g^−1^, respectively. These fast kinetics revealed that GO(0.25)/MR-500 is beneficial in shortening the adsorption cycle time for potential practical applications.

Apart from fast adsorption kinetics, the CO_2_/N_2_ selectivity is also a very important property of adsorbent for CO_2_ capture. Figure 7d shows the N_2_ adsorption isotherm and CO_2_/N_2_ selectivity of GO(0.25)/MR-500. The adsorption capacity of N_2_ is very low, only 0.492 mmol·g^−1^ at 298.15 K and 500 kPa. Highest CO_2_/N_2_ selectivity of 58 was reached at 20 kPa, while the CO_2_/N_2_ selectivity gradually decrease to 39 and 19 at 100 kPa and 500 kPa, respectively, which is still at a high level for high-pressure CO_2_ adsorption compared with the porous carbons reported previously. Surface-doped N, especially for pyrrolic-N, offer abundant sites for high selective adsorption for CO_2_ at low pressure.

**Figure 8:**
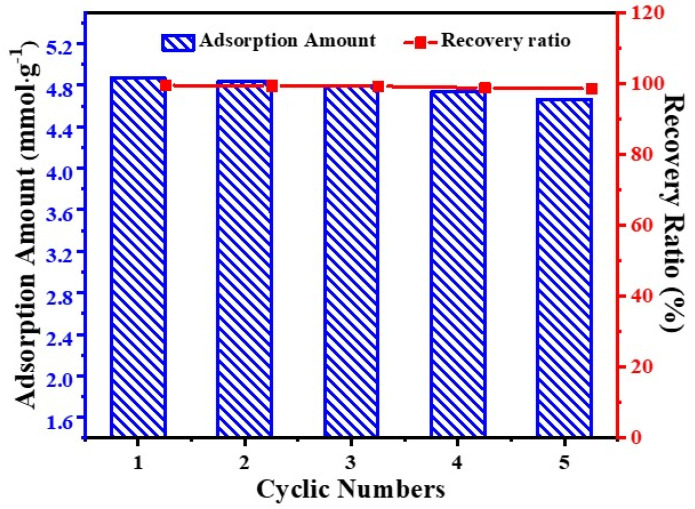
Cycling of CO_2_ adsorption by GO(0.25)/MR-500 at 298.15 K and 500 kPa.

The cyclic performance of adsorbent are very important features for CO_2_ adsorption in practical industrial application. To study the stability and recyclability of GO(0.25)/MR-500 in CO_2_ adsorption, the adsorption was operated at 298.15 K and high pressure (500 kPa), while the in situ regeneration was carried out at 373 K and in a vacuum. It should be note that the adsorbent in our study need to undergo a wide pressure change, which is more likely to destroy the structure of adsorbent compared with the test method of stability reported in literatures. This procedure is also much more similar with the vacuum pressure swing adsorption (VPSA) applied in industry. As shown in Figure 8, GO(0.25)/MR-500 shows excellent stability and the adsorption capacity of CO_2_ could be maintained at 4.66 mmol·g^−1^ with a recovery rate of 98.5% even after five adsorption-desorption cycles.

## 4. Conclusions

Herein, we report the synthesis of high surface area and high N content porous carbons from MR/GO composite with KOH chemical activation. The optimum activation temperature is found to be 500 °C to obtain a porous carbon with a suitable pore structure and the highest BET surface area of 1264 m^2^·g^−1^. Appropriate addition of GO is beneficial to the formation of pores and increases the BET surface area. In addition, high contents of pyridinic-N and pyrrolic-N were formed on the surface of samples after activation, providing abundant adsorption sites for CO_2_. Therefore, GO(0.25)/MR-500 exhibited the highest CO_2_ capture ability of 5.21 mmol·g^−1^ at 298 K and 500 kPa. Detailed studies also revealed the moderate heat of adsorption, fast adsorption kinetics, high selectivity of CO_2_/N_2_ and good recyclability. These results demonstrate the strong potential of MR/GO-derived carbon in this practical application.

## Figures and Tables

**Table 1:** Porous structure, chemical composition and CO_2_ uptake of GO, MR, GO/MR and GO/MR-derived porous carbon materials obtained at different temperatures.

Sample	Textural Properties			Chemical Composition			CO_2_ Adsorbed Amount (mmol·g^−1^)	
	S_BET_ ^a^ (m^2^·g^−1^)	V_p_ ^b^ (cm^3^·g^−1^)	Average Pore Size (nm)	N (wt.%)	C (wt.%)	O (wt.%)	100 kPa	500 kPa
GO	450	2.20	20.8	-	-	-	0.18	0.41
MR	0.99	0.003	-	-	-	-	0.13	0.32
MR-500	204	0.10	3.55	7.11	76.15	16.74	0.19	0.49
GO(0.25)/MR	6.7	0.008	8.50	7.30	67.14	25.56	0.40	0.76
GO(0.25)/MR-400	32	0.06	12.70	6.79	77.93	15.28	0.46	1.05
GO(0.25)/MR-500	1264	0.47	3.90	6.92	80.09	12.99	2.27	5.21
GO(0.25)/MR-600	972	0.67	5.70	6.55	82.42	13.23	1.33	4.29
GO(0.25)/MR-700	790	0.28	3.80	3.42	82.85	13.73	0.87	1.89
GO(0.375)/MR-500	784	0.39	3.16	6.84	81.79	11.37	0.74	2.81

^a^ BET surface area; ^b^ BJH adsorption cumulative volume of pores between 17.000 and 3000.000 Å width.

**Table 2:** Fitting parameters of models for CO_2_ adsorption by the GO(0.25)/MR-500 at different adsorption temperatures.

Model	Fitted Parameters	Temperature (K)			
		298.15	308.15	318.15	323.15
Freundlich	*K_F_* (mmol·g^−1^·kPa^−n^)	0.1623	0.1343	0.0663	0.0548
	n	1.7717	1.7581	1.5050	1.4678
	*χ*^2^ (×10^−4^)	329.9	205.6	256.8	166.5
	*R* ^2^	0.9912	0.9924	0.9890	0.9916
Langmuir	*Q_max_* (mmol·g^−1^)	5.1950	4.4054	3.9498	3.6210
	*K_L_* (kPa^−1^)	0.0041	0.0040	0.0025	0.0022
	*χ*^2^ (×10^−4^)	44.2	68.6	46.2	24.4
	*R* ^2^	0.9988	0.9975	0.9980	0.9988
Redlich–Peterson	*a_R_* (kPa^−bR^)	0.0212	0.0370	0.0033	0.0031
	*K_R_* (mmol·g^−1^·kPa^−n^)	0.0411	0.0391	0.0182	0.0158
	*b_R_*	0.7928	0.7262	0.9600	0.9551
	*χ*^2^ (×10^−4^)	6.3	11.7	55	29
	*R* ^2^	0.9998	0.9996	0.9976	0.9985

## Data Availability

Data are contained within the article.

## Supplementary Materials

The following are available online. Section S1.1: Detailed experimental procedures for CO_2_ adsorption. Figure S1: Diagram of the experimental apparatus for CO_2_ adsorption. Figure S2: N_2_ adsorption-desorption isotherms and pore size distributions of GO/MR derived nitrogen-rich porous carbon materials with different adding amount of GO activated at 500. Figure S3: Wide scan XPS spectra of s GO(0.25)/MR and GO(0.25)/MR derived nitrogen-rich porous carbon materials obtained at different activation temperature. Figure S4: Langmuir and Freundlich isotherm models on experimental CO_2_ adsorption at different temperature for GO(0.25)/MR-500. Figure S5: Detail view of CO_2_ adsorption kinetics at 298.15 K for GO(0.25)/MR-500 (the adsorption amount rang: 4.4–4.9 mmol·g^−1^). Table S1: Adsorption thermodynamic parameters of GO(0.25)/MR-500. Table S2: Adsorption thermodynamic parameters of GO(0.25)/MR-500 at different CO_2_ adsorption capacities. Table S3: Fitted parameters of CO_2_ adsorption kinetic models on the GO(0.25)/MR-500 at 298.15 K.

## Sample Availability

The samples are available from the authors.
