# Supplementary material for: Nitrogen-Doped Porous Carbon Materials Derived from Graphene Oxide/Melamine Resin Composites for CO2 Adsorption
Source: Molecules. 2021 Aug 31;26(17):5293. doi: 10.3390/molecules26175293 (PMC8434559; doi:10.3390/molecules26175293)
Supplement: Supplementary file 1 [file molecules-26-05293-s001.zip › molecules-1328990-supplementary.pdf]

Supplementary Information

Nitrogen-Doped Porous Carbon Materials Derived from Graphene Oxide/Melamine Resin Composites for CO_2 Adsorption

Like Ouyang, Jianfei Xiao, Housheng Jiang, Shaojun Yuan *

Low-carbon Technology & Chemical Reaction Engineering Lab, College of Chemical Engineering, Sichuan University, Chengdu 610065, China

* Corresponding author: ysj@scu.edu.cn (S.J. Yuan), Tel./Fax: +86-28-85405201

S1 Experimental Section

S1.1 Calculation method for CO_2 adsorption capacity

Before the experiment, the adsorption system was evacuated with a vacuum pump until the pressure value was less than 0.2 kPa in the desired temperature. Then, the desired amount of adsorbate gas was supplied to the pressure tank and the molar number was calculated by the van der Waals equation at the equilibrium pressure and temperature. Finally, the adsorbate gas was admitted into the adsorption tank until the pressure indicator remains unchanged in 30 min, and it is considered that the adsorption equilibrium is achieved. The equilibrium pressure and temperature were recorded. The high purity helium as calibration gas, using the **Equation (S1)** to calculate the adsorption tank free space volume (V_{fs}). **Equation (S2)** can be used to calculate the adsorption capacity of CO_2 gas (purity of 99.99 %) when it first enters the adsorption tank to achieve equilibrium. As long as the steps are repeated, the amount of adsorption under different pressures can be measured by **Equation (S3)**.

$$V_{fs}=\frac{V_p(v_{He\text{-}pfs}-v_{He\text{-}p})}{v_{He\text{-}p}} \tag{S1}$$

$$n_i=\frac{V_p}{v_{p,i}m}-\frac{V_p+V_{fs}}{v_{pfs,i}m} \qquad i=1 \tag{S2}$$

$$n_i=n_{i-1}+\frac{V_p}{v_{p,i}m}+\frac{V_{fs}}{v_{pfs,i-1}m}-\frac{V_p+V_{fs}}{v_{pfs,i}m} \qquad i=2,3,4\ldots \tag{S3}$$

Where V_p (63.55 mL) and V_a (12.45 mL) are volumes of pressure tank, adsorption tank respectively; V_{fs} (mL) is the effective free space volume after

placing the adsorbent; v_{He-p} (mL/mol) is the molar volume of helium gas filled with the pressure tank; v_{He-pfs} (mL/mol) is the molar volume of adsorption tank when helium reaches equilibrium of adsorption; $\mathrm{v}_{p,i}$ (mL/mol) is the molar volume of CO_2 gas filled with the pressure tank; $v_{pfs,i}$ (mL/mol) is the molar volume of adsorption tank when CO_2 gas reaches equilibrium of adsorption; $m(g)$ is the mass of adsorbent and i is the number of adsorption equilibrium.

S2 Results and Discussion

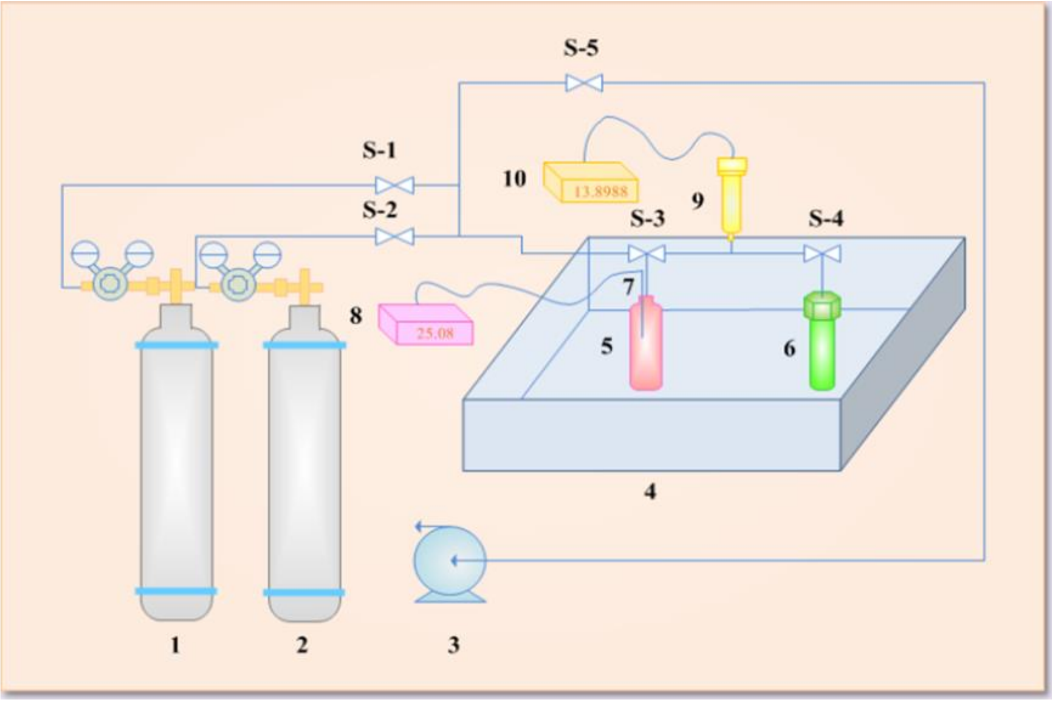

Figure S1. Diagram of the experimental apparatus: (1) helium tank, (2) carbon dioxide tank, (3) vacuum pump, (4) thermostatic bath box, (5) pressure tank, (6) adsorption tank, (7) temperature sensor, (8) temperature monitor, (9) pressure sensor and (10) pressure monitor.

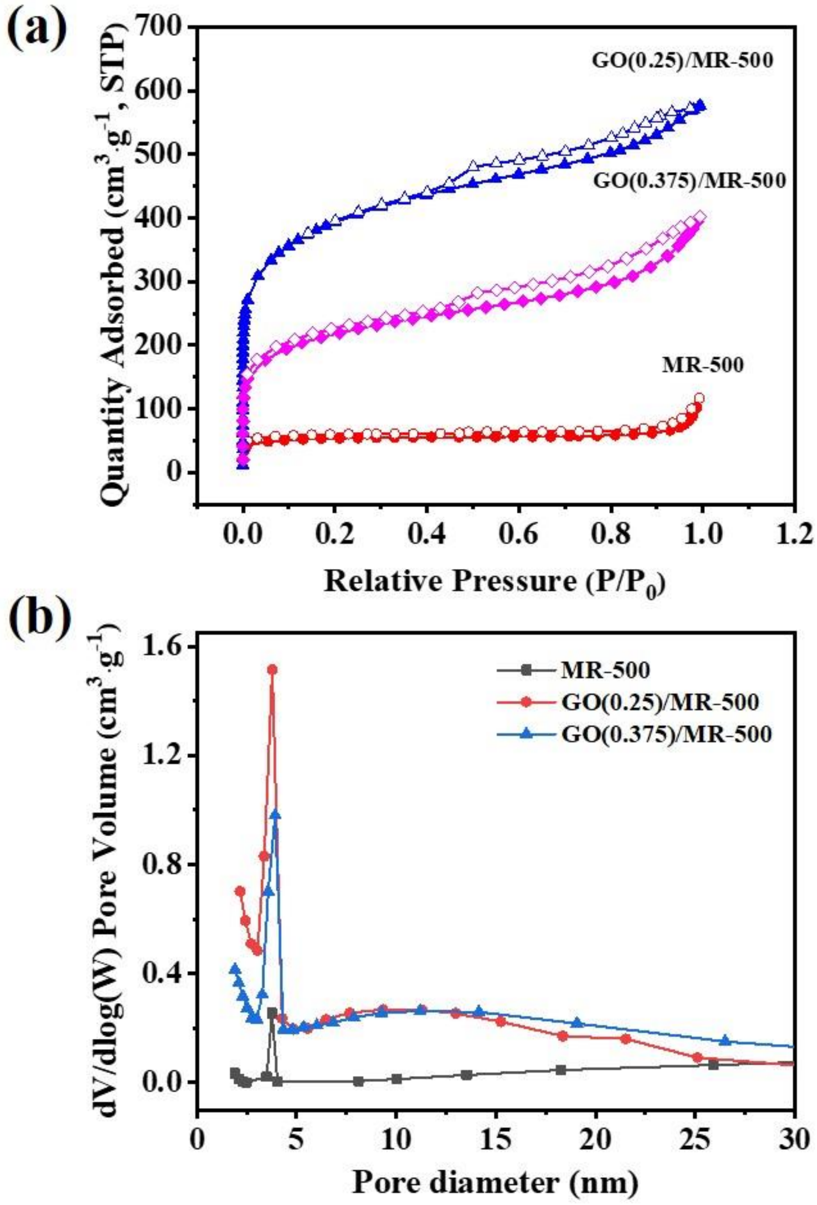

Figure S2. (**a**) N_2 adsorption-desorption isotherms and (**b**) Pore size distributions of GO/MR derived nitrogen-rich porous carbon materials with different adding amount of GO activated at 500.

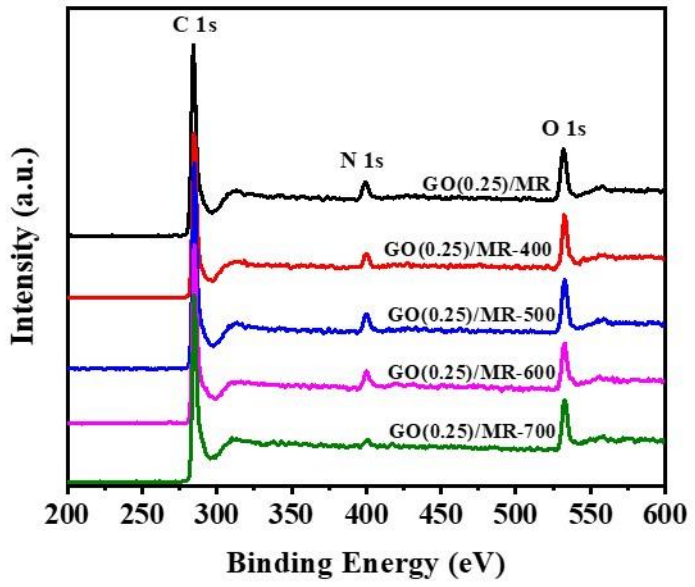

Figure S3. Wide scan XPS spectra of s GO(0.25)/MR and GO(0.25)/MR derived nitrogen-rich porous carbon materials obtained at different activation temperature.

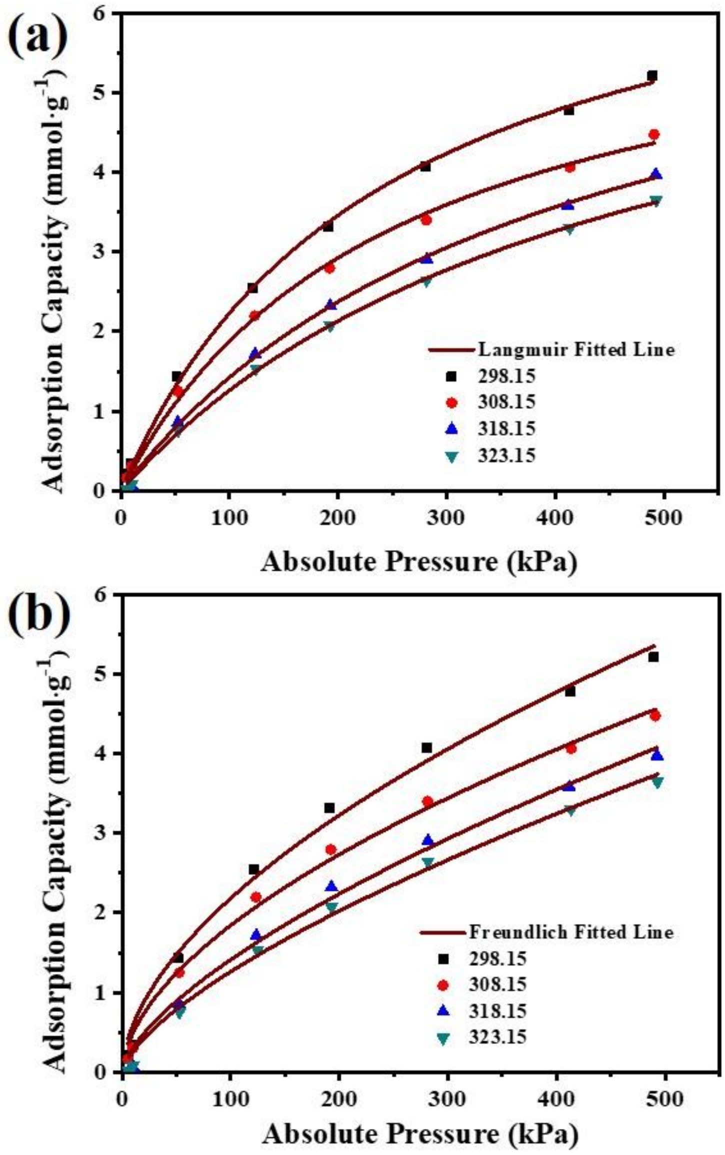

Figure S4. (**a**) Langmuir and (**b**) Freundlich isotherm models on experimental CO_2 adsorption at different temperature for GO(0.25)/MR-500.

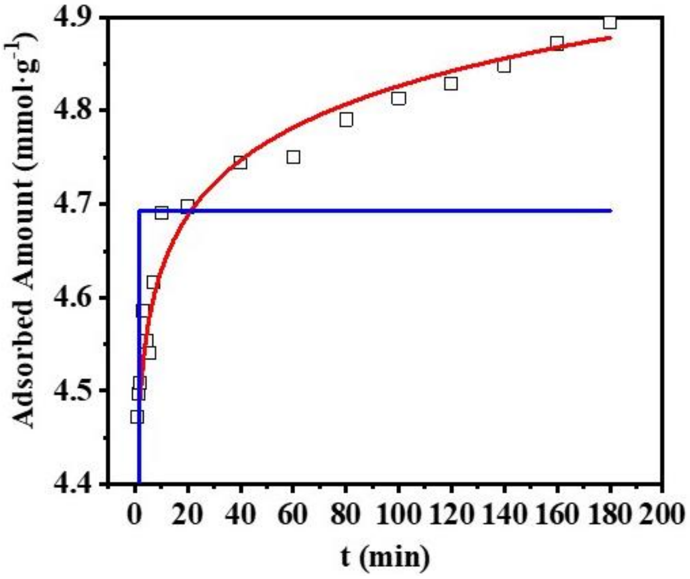

Figure S5. Detail view of CO_2 adsorption kinetics at 298.15 K for GO(0.25)/MR-500 (the adsorption amount rang: 4.4-4.9 mmol·g^{-1}).

Table S1. Nitrogen species of the GO(0.25)/MR precursor and GO(0.25)/MR derived porous carbon materials derived from the N1s XPS spectra.

Sample	Nitrogen (wt%)				
	Pyridinic-N	Amide/Imide-N	Pyrrolic-N	Quaternary-N	Oxidized-N
GO(0.25)/MR		71.9		28.1	
GO(0.25)/MR-400	42.5		45.1	9.1	3.3
GO(0.25)/MR-500	28.3		56.6	9.6	5.5
GO(0.25)/MR-600	25.4		55.2	11.3	8.1
GO(0.25)/MR-700	29.8		54.3	16.0	

Table S2. Adsorption thermodynamic parameters of GO(0.25)/MR-500 at different CO_2 adsorption capacities.

CO_2 uptake ($mmol\cdot g^{-1}$)	ΔH ($kJ\cdot mol^{-1}$)	ΔS ($J\cdot mol^{-1}\cdot K^{-1}$)
0.5	-29.88	183.9
1.5	-26.81	180.7
2.5	-24.99	180.3
3.5	-24.43	179.1

Table S3. Fitted parameters of CO_2 adsorption kinetic models on the GO(0.25)/MR-500 at 298.15 K.

Sample	Models	Fitted Parameters					
		k_F (min^{-n})	k_L (min^{-1})	Q_e ($mmol\cdot g^{-1}$)	n	χ^2($\times 10^{-3}$)	R^2
GO(0.25)/MR-500	Fickian diffusion	2.11	-	4.87	0.0181	1.46	0.999
	LDF	-	4.68	4.69	-	18.21	0.985
